# Do Our Means of Inquiry Match our Intentions?

**DOI:** 10.3389/fpsyg.2016.01048

**Published:** 2016-07-19

**Authors:** Yaacov Petscher

**Affiliations:** Florida Center for Reading Research, Florida State University, TallahasseeFL, USA

**Keywords:** quantile regression, psychological well-being, reading achievement, regression, conditional median modeling, conditional mean modeling

## Abstract

A key stage of the scientific method is the analysis of data, yet despite the variety of methods that are available to researchers they are most frequently distilled to a model that focuses on the average relation between variables. Although research questions are frequently conceived with broad inquiry in mind, most regression methods are limited in comprehensively evaluating how observed behaviors are related to each other. Quantile regression is a largely unknown yet well-suited analytic technique similar to traditional regression analysis, but allows for a more systematic approach to understanding complex associations among observed phenomena in the psychological sciences. Data from the National Education Longitudinal Study of 1988/2000 are used to illustrate how quantile regression overcomes the limitations of average associations in linear regression by showing that psychological well-being and sex each differentially relate to reading achievement depending on one’s level of reading achievement.

## Introduction

The field of psychological science is a multifaceted discipline, touching on everything from emotion and intelligence to academic achievement and morality. Quantitative methods for studying phenomena starts with observations, creating questions and formulating hypotheses about causality or individual differences, gathering data, and finally analyzing and reporting results for evaluation (Cronbach, 1957). Despite the relative straightforwardness of the scientific method, the choice of an analytic framework frequently appears to be anything but. Whether choosing among classes of regression models, using observed or latent variables, selecting critical hypothesis test values and effect size metrics, one seemingly creates a *choose-your-own-adventure* type of picture in the mind based on *if-then* statements to select appropriate techniques to analyze data. Yet at the end of this type of mental exercise, a frequent conclusion is the choice of a methodology that is fundamentally rooted in conditional means modeling. Simply stated, a conditional mean model, such as a simple linear regression, estimates an expected average outcome value for a given value of one or more predictors.

The prevalence of conditional means models in most educational and psychological graduate training programs as well as the density of use in the literature has, on some level, conditioned us to expect that such models are the expected norm. The principal goal in this paper is to introduce the reader to a conditional median framework, which is largely unknown and seldom used in psychological methods. This introduction is framed by addressing the issue of why the mean is prevalent and what the impact of the mean is on our understanding of individual differences in research. The conditional median framework is shown to be complementary to a conditional mean framework to the point that regression analysis in the former framework can illuminate more comprehensive relations compared to the latter framework.

### Why the Mean?

As the header question asks, why is the mean so important? Speelman and McGann (2013) noted that the mean is frequently used because introductory textbooks in psychology and methods, and by proxy other introductory text books in related areas, give a type of elevated status to the mean compared to other measures of central tendency for a data set. The authors noted specifically that, “This mean value is then discussed as representing the average performance of the group, as if this value provides a representative substitute for the group’s data.” (p. 1). Consequently, the statistical moment *mean* is often used synonymously with *descriptive statistic* or *summary statistic* when synthesizing data of some observed phenomenon. Although the authors do not advocate abandoning the use of such a fundamental measure of central tendency, they raise valid concerns about its use as a panacea for understanding data relations. In many areas of psychological and educational research including memory experiments (Anderson and Schooler, 1991), response time measurement, and the development of early literacy skills (Catts et al., 2009), the mean can be insufficient to capture individual differences either due to the presence of measurement artifacts, such as floor or ceiling effects (Catts et al., 2009), or to phenomenon such as the power law of learning (Anderson and Schooler, 1991). Such inadequacies led Speelman and McGann (2013) to suggest that using the mean suppresses important individual differences in data analyses.

### Implications of the Mean on our Models

In order to better understand how using means-based modeling may color the way we think about individual differences research, it is useful to evaluate common goals of data analysis. Statistical data analysis has two philosophical goals of prediction and understanding (Shmueli, 2010). A fundamental reason statistical models are rooted in a conditional means framework is that data analyses are used, in part, for *prediction* (Shmueli, 2010). When a goal for predictive modeling is to generate reliable estimates for projecting future behaviors or events, the mean is often needed for the estimation process to be done correctly. Predicting future values on an outcome is not the sole goal of statistical models. Even though a statistical model is inherently inferential, a researcher does not always use the model to then generate anticipated estimates for the future. Rather, scientists aim to *understand* what other variables explains individual differences in the selected outcome(s). Although prediction and understanding have unique and common properties, they also become easily conflated in practice (Shmueli, 2010).

Whether statistical methods are used for prediction or understanding, research typically starts with a broad question about the relation between observed phenomena in the vein of, “What is the relation between *X* and *Y*?” Although the question is broad, our statistical models inherently refine that question to, “On average, what is the relation between *X* and *Y*?” In some cases the question of average relations may be of importance, but in many conditions our questions are not just about average relations but the association between observed phenomena across all levels of an outcome of interest. For a question such as, “What is the relation between teacher instruction and student achievement?” average relations only scratch the surface between what may be happening in the classroom and how students perform on standardized measures of cognitive achievement (Connor et al., 2007). A more comprehensive operationalization of this question could be, “What is the relation between teacher instruction and student achievement for students with varying levels of achievement?” Traditional conditional means models are not well suited to such questions. Researchers often try to contextualize the outcomes by evaluating relations at other, somewhat arbitrary points in the distribution such as one standard deviation below the mean, or by splitting the outcome variable into multiple groupings (e.g., deciles). Such approaches, however, are limited due to a restriction of range in the outcome based on the grouping, as well as reduced power to detect statistically significant relations due to reduced sample size.

### Conditional Median Modeling

An alternative framework that overcomes the limitations of conditional means modeling is conditional median modeling. The median is a well-known measure of central tendency, and a recognized property of the median is that when data are normally distributed the median is equal to the mean. A special case of conditional median models, known as quantile regression (Koenker and Bassett, 1978), allows for an analysis of relations among variables conditional on other points of the outcome other than the mean or median. Quantile regression allows a user to specify multiple quantiles (where a quantile is conceptually similar to a percentile), simultaneously estimate the relations among the variables at each quantile, and produce coefficients, standard errors, *t*-values, and *p*-values for each of the intercept and slope parameters at each quantile. A unique property of quantile regression is that when sample data are normally distributed, results from a quantile regression at the 0.50 quantile (i.e., approximately the 50th percentile) will be identical to that of a conditional means analysis via linear regression.

Quantile regression has been used more widely in econometrics to explore such things as March Madness and bracketology in college basketball (Koenker and Bassett, 2010) and the relation between foreign aid and corruption (Okada and Samreth, 2012), as well as wage inequality in sociology (Killewald and Bearak, 2014) and in isolated instances in educational and psychological research (e.g., Catts et al., 2009; Petscher and Kim, 2011; Petscher and Logan, 2014), but is largely an unknown and under-utilized methodology.

The form of quantile regression in the conditional median model is quite close to that of ordinary least squares (OLS) in the conditional mean model. Consider a traditional expression of a simple linear regression

$$Y_{i}=\beta_{0}+\beta_{1}X_{i}+\epsilon_{i}$$

where Y_i_ is the score on dependent variable *Y* for student *i*, β_0_ is the intercept, β_1_ is a slope, X_i_ is the score for student *i* on independent variable *X*, and ε_i_ is a residual term. Values for the intercept and slope terms are estimated by a loss function

β^=argmin⁡β∈RKΣi=1n(yi−xi′β)2

where 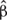 is solved by finding the value that minimizes the sum of the squared residuals. In quantile regression, the expression of a simple linear regression is nearly identical to Equation 1 with

$$Y_{i}=\beta_{0\tau }+\beta_{1\tau }X_{i}+\epsilon_{i\tau }$$

where the included terms are a dependent variable for an individual, an intercept, slope, value on an independent variable, and a residual. The difference between this equation and Eq. 1 is the inclusion of τ which represents the quantile of interest where the intercept, slope, and residual are estimated. For a given τ, the intercept and slope coefficients can be estimated with a formula similar to the loss function used for OLS in Eq. 2 with

β^τ=⁡argminβ∈RKΣi=1n(ρτ(yi−xi′β))

This expression includes both 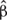
_τ_ where the coefficient is estimated where the distance of the residual is being minimized at a specified quantile of 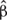 rather than the sum of squared residuals, and ρ_τ_ which is the weighted distance used to calculate the objective function in the minimization algorithm.

Where the conditional median framework via quantile regression maintains benefits over the conditional mean framework via OLS is both in the algebraic expressions and assumptions. In OLS, distributional assumptions are made about ε_i_ being identically, independently, and normally distributed with a mean of 0 and variance of σ^2^. No such assumptions applied to the residual in quantile regression, implying that quantile regression is a robust approach to data that present with non-normal distributions. Despite the lack of specific assumptions on normality and linearity, linear quantile regression is a parametric analysis. The advantage of parametric quantile regression compared to OLS is that although both techniques imply a distribution for the dependent variable, OLS’ kernel density functions are not as well aligned to the actual density as the quantile regression density function (McMillen, 2012). The better alignment between estimated and actual densities for quantile regression is due to the implied distribution changing by varying amounts across the distribution. That is, because different slope coefficients are estimated along the conditional distribution, quantile regression can better reflect the actual conditional distribution of the dependent variable by estimating unique coefficients. Conversely, the OLS’ density function changes only by a constant amount due to its estimate of one intercept and slope coefficient, thus leading to a less accurate reflection of the data.

From a more applied perspective, parametric quantile regression can be used to specifically model the inherent variability within one’s dataset. Whereas OLS creates a single curve or line of fit to characterize the conditional mean relation between dependent and independent variables, quantile regression creates a series of lines at each quantile of the dependent variable conditional on a value of the independent variable. Just as OLS leverages a full sample to produce an estimated intercept and slope coefficient based on the conditional mean, so does quantile regression leverage a full sample to produce an estimated intercept and slope coefficient conditional on the quantile of interest. In this way, estimates from a quantile regression imply a full distribution of conditional values for the dependent variable, and it is possible to more fully understand complex relations between variables without sacrificing statistical power (McMillen, 2012). Because the full sample is used with asymmetric weighting across quantiles, quantile regression is not akin to creating deciles on an outcome but rather uses the loss function for estimation.

In a recent application of quantile regression, Petscher and Logan (2014) used the High School and Beyond dataset to show that the minority gap in math achievement in 10th grade is conditional on math performance, such that at the mean of math achievement Minority students scored 5 points lower than White students; however, for students with lower math achievement (i.e., 0.10 quantile) the minority achievement gap was 3 points and for students with higher math achievement (i.e., 0.90 quantile) the gap was only 2 points. Although this example highlights the potential use of quantile regression, the remainder of this article walks the reader through a comparison of using conditional mean and median modeling analyses via OLS and quantile regression.

## Method

### Sample

Subjects were drawn from the National Education Longitudinal Study of 1988/2000 (NELS; USDE National Center for Education Statistics, 2004)^1^ The NELS survey began in 1988 with eighth-grade students and followed them intermittently with final follow-up in 2000. For the purpose of these analyses, the inclusion criteria were to retain students who had available data on sex, grade 8 item-level data on psychological well-being, and grade 12 standardized reading achievement data. In the final data set, a total of 8,649 participants were included; 53% were female.

### Analysis

Two sets of analyses were conducted. First, grade 8 psychological well-being was used as a predictor of grade 12 reading achievement. The second analysis evaluated differences in reading achievement between males and females. In both cases, OLS and quantile regression models were estimated and results were compared. To facilitate ease in the interpretation of results, the well-being and reading achievement data were standardized within the sample as a *z*-score, allowing model coefficients to be interpreted approximately as correlations.

## Results

### Descriptive Statistics and Correlations

The average grade 12 reading score from the NELS was 51 0.19 (*SD* = 9.79, Minimum = 29, Maximum = 68). Other statistics suggested that the distribution of data presented with little skew (–0.30) but some kurtosis (–0.95). The psychological well-being from eighth-grade showed a mean of 39.36 (*SD* = 5.77, Minimum = 3, Maximum = 52). Similar to the reading data, psychological well-being was generally normally distributed with some skew present (–0.48) but stronger kurtosis compared to reading (1.25). Reading scores were positively correlated with psychological well-being [*r*(8647) = 0.26, *p* < 0.001] and sex [*r*(8647) = 0.18, *p* < 0.001); well-being and sex were negatively, but weakly associated [*r*(8647) = –0.12, *p* < 0.001].

### Analysis 1

**FIGURE 1:**
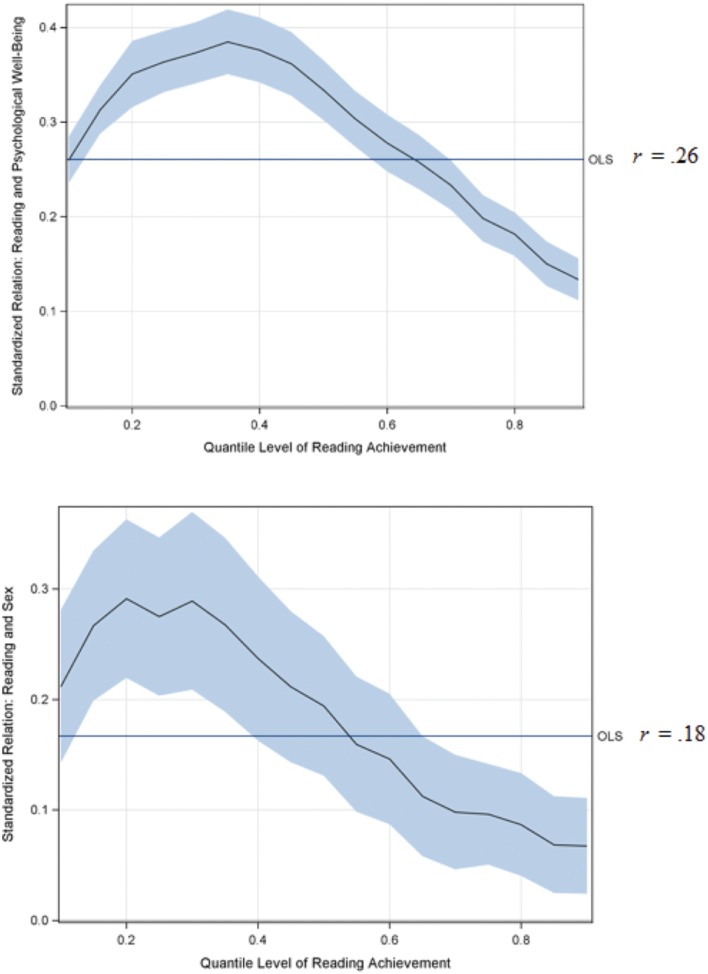
**Quantile and linear regression results for the prediction of grade 12 reading achievement from grade 8 psychological well-being **(top)** or sex **(bottom)**.** The *x*-axis is the quantile of reading achievement, the *y*-axis is the coefficient for the standardized relation between reading achievement and the independent variable, the ordinary least squares (OLS) reference line is the standardized relation between reading achievement and the independent variable from an OLS regression, the black line represents the coefficient value for the *x*-axis, the shading around the black line is the confidence interval around the coefficient. Note that the OLS value is one value and not a plot as in the quantile regression.

All statistical models were estimated using SAS with the GLM procedure for the OLS analysis and the QUANTREG procedure for the quantile regression analysis. To summarize results, a graphical depiction of the comparison between the two procedures for the relation between reading achievement and psychological well-being are displayed in **Figure 1.** The plot contains several components: (1) the *x*-axis is the quantile of grade 12 reading achievement, (2) the *y*-axis is the range of estimated coefficients for the relation between well-being and reading achievement, (3) a horizontal reference line representing the estimated well-being coefficient estimated from OLS, and (4) the line with shading represents the estimated coefficient for well-being at each quantile (line) along with a 95% confidence interval (shading). A comparison between quantile and OLS regressions reveals important differences. Using OLS regression, the estimated correlation between well-being and reading achievement was *r* = 0.26 (*p* < 0.001). Research studying this type of relation would normally conclude that a relatively weak association exists between the two variables with an average of 7% of the variance in grade 12 reading achievement explained by grade 8 psychological well-being. Conversely, the quantile line demonstrates that weak to moderate associations exists between well-being and reading achievement exists depending on one’s level of reading achievement. At high levels of reading (e.g., 0.80 quantile) psychological well-being is a weak predictor of one’s reading scores 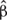 = 0.18, *p* < 0.001), yet at low levels of reading scores (e.g., between the 0.20 and 0.45 quantiles) well-being was a more robust predictor of reading with coefficients ranging from 0.35 to 0.39 (*p* < 0.001). That is, although the OLS regression suggested that, on average, 6% of the variance in reading scores was explained by well-being, quantile results suggested that anywhere between 2% of the variance and 15% of the variance in reading is explained by psychological well-being dependent on one’s level of reading achievement. More succinctly, at higher levels of reading, psychological well-being is weakly predictive of such achievement; conversely, low reading scores were more strongly predicted by low psychological well-being. Note that in this example the OLS value of 0.26 approximated that of the quantile analysis at the 0.50 quantile (0.33, *p* < 0.001), yet the mean and the median were not identical due to psychological well-being presenting with larger kurtosis (i.e., 1.25). It is further important to recognize that OLS regression cannot estimate the value of 0.18 at the 0.80 quantiles, nor the 0.39 estimate at the 0.35 quantile because that model is conditional on the mean. Suppose one were interested in trying to estimate the relation for students at the highest levels of reading achievement. First, if one isolated only individuals at the 80th percentile, the sample size reduces from 8,649 to 87 individuals. Moreover, there is a severe restriction of range such that the mean standardized reading score is 0.97 with a standard deviation of 0.007. An applied OLS regression model to these data results in model estimated standardized slope coefficient of –0.000009 (*p* = 0.991) because there is no variability in the outcome a specific value of the outcome. Rather, quantile regression leverages the full data to estimate the relation *conditional* on different points of the distribution and not in isolation as in OLS regression.

### Analysis 2

Results from the analysis of sex differences in reading achievement are also shown in **Figure 1.** Note that in this model a dichotomous predictor was used (i.e., female/male) compared to the continuous predictor in Model 1. From a substantive perspective, the interpretation of the model coefficients is one of differences between males and females in standardized units. In the linear regression analysis, it was observed that on average females scored higher than males by 0.18 standard deviation units (*p* < 0.001). Similarly, the quantile regression at the 0.50 quantile showed a nearly identical gap in reading performance (0.19 standard deviation units; *p* < 0.001), reflecting that the reading achievement measure was normally distributed. The quantile regression results provided a different picture from the linear regression by demonstrating that at lower levels of reading performance, females outperformed males by up to 0.30 standard deviation units (*p* < 0.001), but at high levels of reading achievement the two sexes became less distinguished in reading performance.

## Discussion

Questions about the purpose of and over-reliance on the mean as a summary statistic have been raised in the literature as of late. Both Balota and Yap (2011) and Speelman and McGann (2013) noted that the mean serves a valuable purpose in summarizing data but that at times the mean can mislead the reader in terms of a descriptive summary and in our inferential modeling. Speelman and McGann (2013) laid out assumptions made about means-based modeling and the potential implications of the violations of those assumptions on data interpretations. Balota and Yap (2011) encouraged researchers to plot their distributions through a variety of methods to better understand the nature of one’s data for individual differences. In this manuscript, both sets of authors’ works are extended to provide a potential solution that overcomes the use of the mean, namely, a conditional median framework with quantile regression. The foundational algorithms that underlie quantile regression are similar to that of the conditional means models, yet by estimating individual intercept and slope coefficients, researchers may gain comprehensive insight into summarize phenomena while still conducting hypothesis testing. The recommendations by Balota and Yap (2011) are a useful start, and as with all research projects it is important to first understand the nature of data distributions. By extending the exploratory, descriptive solution to an inferential solution, it is possible to advance our scientific method.

By leveraging a large publically database, it was demonstrated that conditional means models used in most research studies may not comprehensively serve to explain the relations among observed phenomena. In both examples, the quantile regression model demonstrated that eighth-grade psychological well-being and sex differentially relate to later reading achievement depending on the child’s actual level of reading performance. Such nuanced relations were not possible using traditional linear regression. These examples highlight that quantile regression may be a useful approach as both a primary analysis or as a complementary technique to traditional models. Furthermore, the example of the dichotomous predictor could be generalized to any dichotomy, including a treatment effect indicator from randomized controlled trials, in the regression analysis.

In returning to the reasons why statistical modeling is done, whether we are interested in prediction or understanding, both reasons are aligned to an overarching goal of answering the simple question of, “What is the relation between *X* and *Y*?” By using this quantile regression as a special case of conditional median modeling, it may be possible to better answer this question and illuminate individual differences as well as expand our notion of testing for heteroscedastic treatment effects (e.g., Venturini et al., 2014; Wanzek et al., in press) without reducing power, restricting score ranges, or minimizing samples as when occurs by other workarounds to the limitations posed by conditional means models.

## Author Contributions

The author confirms being the sole contributor of this work and approved it for publication.

## Conflict of Interest Statement

The author declares that the research was conducted in the absence of any commercial or financial relationships that could be construed as a potential conflict of interest.

## Footnotes

1 http://www.icpsr.umich.edu/index.html.
